# ROS‐driven cellular methane formation: Potential implications for health sciences

**DOI:** 10.1002/ctm2.905

**Published:** 2022-07-15

**Authors:** Frank Keppler, Leonard Ernst, Daniela Polag, Jingyao Zhang, Mihaly Boros

**Affiliations:** ^1^ Biogeochemistry Group Institute of Earth Sciences Heidelberg University Heidelberg Germany; ^2^ Heidelberg Center for the Environment (HCE) Heidelberg University Heidelberg Germany; ^3^ Max‐Planck‐Institute for Terrestrial Microbiology Marburg Germany; ^4^ Department of Hepatobiliary Surgery and Department of SICU The First Affiliated Hospital of Xi'an Jiaotong University Xi'an Shaanxi China; ^5^ Institute of Surgical Research and Interdisciplinary Excellence Centre University of Szeged Szeged Hungary

## Abstract

Recently it has been proposed that methane might be produced by all living organisms via a mechanism driven by reactive oxygen species that arise through the metabolic activity of cells. Here, we summarise details of this novel reaction pathway and discuss its potential significance for clinical and health sciences. In particular, we highlight the role of oxidative stress in cellular methane formation. As several recent studies also demonstrated the anti‐inflammatory potential for exogenous methane‐based approaches in mammalians, this article addresses the intriguing question if ROS‐driven methane formation has a general physiological role and associated diagnostic potential.

For a long time, biological methane (CH_4_) formation was considered to exclusively be produced enzymatically by methanogenic *Archaea* – so called methanogens – living in oxygen‐free environments. Recent discoveries demonstrated that aerobic life, including both eukaryotes and bacteria, does also produce methane.^1^, ^2^ However, the underlying mechanism(s) remained unclear. A recent study by Ernst et al.^3^ proposed a mechanism that might be common across all living organisms. Metabolic activity, especially under the influence of oxygen, leads to the formation of reactive oxygen species (ROS) in cells, which include superoxide ion, hydrogen peroxide (H_2_O_2_) and hydroxyl radicals (^.^OH).  In interaction with the essential element iron, the Fenton reaction takes place – a reaction between reduced iron and H_2_O_2_ that leads to the formation of highly reactive tetravalent iron compounds and hydroxyl radicals. The latter molecules drive the cleavage of a methyl radical from methylated sulphur and nitrogen compounds, e.g. the amino acid methionine, dimethyl sulphoxide or trimethylamine. In a subsequent reaction of the methyl radical with a hydrogen atom, methane is finally formed. The observation of ROS‐driven formation of methane was confirmed in over 30 model organisms, ranging from bacteria and archaea to yeasts, plant cells and several human cell lines. Additional oxidative stress, triggered by physical and chemical factors such as higher ambient temperatures or the addition of ROS‐forming substances, also led to an increase in methane formation in the examined organisms (Figure 1). In contrast, the addition of antioxidants and the scavenging of free radicals reduced the formation of methane – an interaction that probably controls the formation of methane in organisms. Because the Fenton reaction – which drives the formation of hydroxyl radicals – is a universal feature of aerobic life, we speculate that fluctuating methane levels can be detected in all eukaryotic organisms at different metabolic stages but also might be a hallmark for different pathophysiology.

**FIGURE 1:**
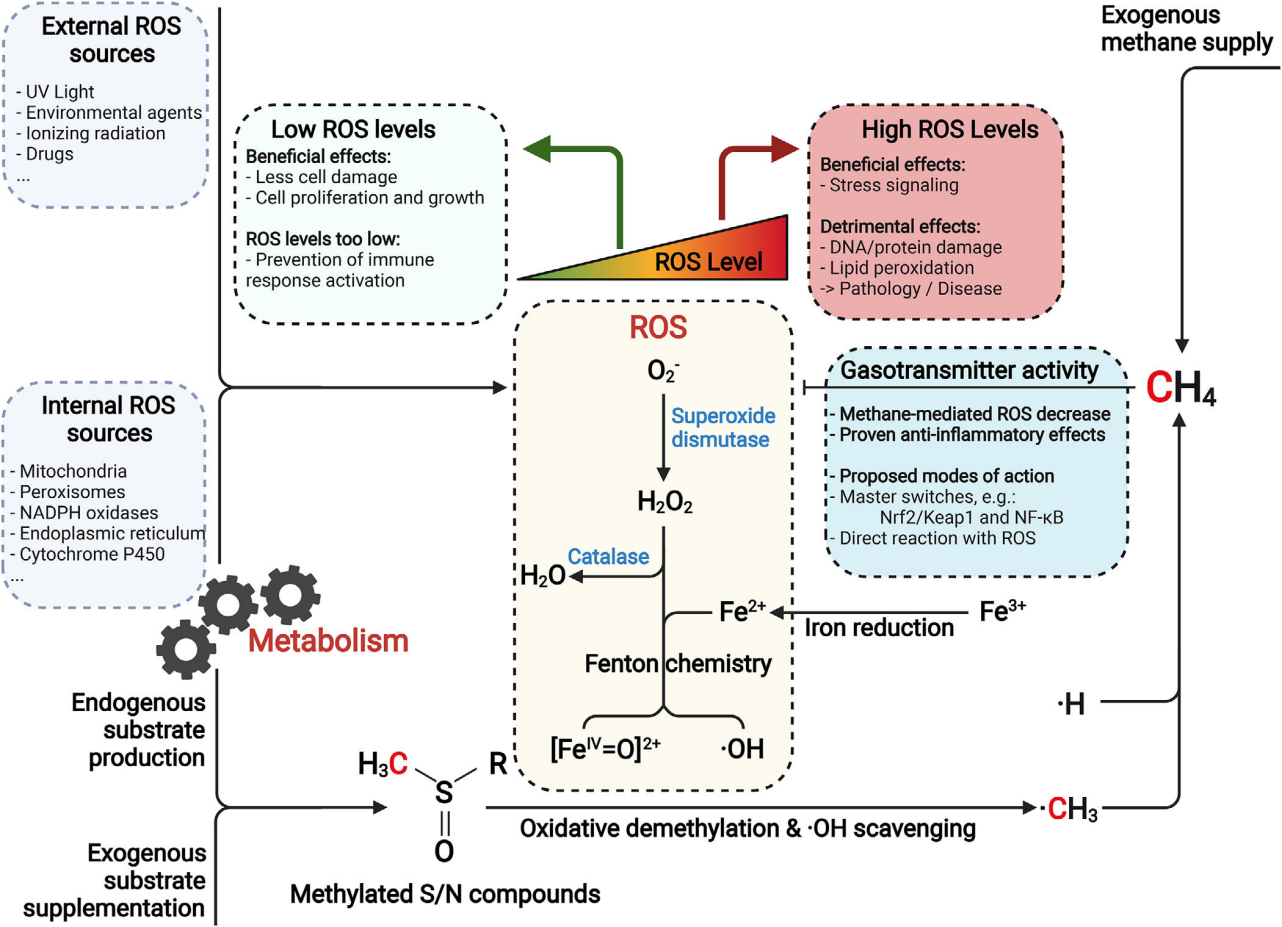
Potential role of ROS‐driven methane formation and consumption in cellular metabolism and physiology. It is proposed that methylated S‐/N‐compounds act as ∙OH scavenger, while the thereby produced methane acts as gasotransmitter indicating elevated ROS levels

From here on we will discuss the potential significance of these results for clinical and health sciences. First, we outline the general role of ROS in mammalian cells and what consequences this has for our understanding of cellular methane formation in mammalians. Initially, the occurrence of ROS and its presumed toxicity has been considered as the paradox of aerobic life. Today, it is clear that ROS also play multiple beneficial roles in the cellular functioning of aerobic organisms.^4^, ^5^, ^6^ ROS are required for many important signalling reactions but are also toxic by‐products of aerobic metabolism, but as always the dose makes the poison. Thus, aerobic cells have installed many antioxidative systems trying to keep the level of ROS in a non‐toxic range. Frequently increased oxidative stress also classified as distress leading to an overproduction of ROS has been suggested to be involved in a wide variety of age‐related disorders, degenerative processes, syndromes, and many diseases such as cancer, heart attacks, atherosclerosis, chronic inflammatory diseases, arteriosclerosis, ischaemia/reperfusion injury, Alzheimer's dementia and Parkinson's disease. Finally, ROS might be the key for understanding the process of aging itself. As it has now been shown^3^ that ROS induce methane formation in aerobic cells, a range of novel research opportunities for medical sciences emerge. Thus, it can be easily envisaged that methane might serve as a read‐out for enhanced ROS levels in humans. The concentration of H_2_O_2_ in the normal cytoplasm, mitochondrial matrix, and endoplasmic reticulum (ER) lumen varies by several orders of magnitudes (from 80 pM to 700 nM),^7^ which implicates that methane might be formed at largely fluctuating levels in different organelles. Therefore, we speculate that methane might also serve as a hallmark of organelle damage like ER stress. The observation that the probability of elevated breath methane levels increases with advanced age might be an indication of the age‐related increase of systemic inflammation accompanied with enhanced ROS levels.^8^ Long‐term monitoring studies of breath methane provide further evidence that abrupt deviations in breath methane levels from baseline are linked to immune reactions and inflammatory processes.^9^ In this context, infectious diseases were mostly accompanied by temporary elevated breath methane production. One might further conclude that vaccinations as induced perturbations of the immune system will cause substantial fluctuations in the breath methane level of people indicating individual immune responses and immune states. Thus, monitoring breath methane levels might have great potential for ‘in vivo’ diagnostics. However, the methane breath approach as an indicator for fluctuating ROS levels might only apply when methane formation by methanogenic archaea in the gastrointestinal tract of the subject is relatively low, otherwise the background breath methane level might mask the ROS‐related effects.

Next to ROS, the concentrations of free iron – in the form of iron(II) – and methylated compounds – particularly those where the methyl group is bonded to sulphur and nitrogen compounds, such as methionine or DMSO – might be the other key factors contributing to methane formation and affect the efficiency of ROS consumption. Recent studies showed that iron metabolism represents a key factor in oncology, immunological, neurological and infectious diseases. Harmful oxidative distress could be observed in states of both iron deficiency (anemia) and overload (ferroptosis).^10^ It is conceivable that appropriate iron supplementation is beneficial for the health, which may be associated with its role in contributing to the homeostasis of cellular ROS through methane formation. It is also obvious that the available various methylated compounds will cause different efficiency of methane formation and ROS consumption. The intermediate methyl radical cleaved off from the methylated precursor might also play a role in methylation processes and thus be considered in medicinal biochemistry. To date, several preclinical and clinical studies proved that multi‐methyl drugs such as metformin, trimethylglycine or tetramethylpyrazine exert significant advances in therapeutic effect. Exogenous supplementation of methylated compounds might substantially increase methane formation and ROS consumption. In this context, it is of high interest to discuss the potential role of DMSO as an effective radical scavenger to counteract enhanced oxidative stress induced by ROS. DMSO has already been studied and applied for many decades but its potential beneficial role for medical use has remained highly controversial. However, revisiting and studying the potential role of DMSO for medical research in greater detail might be worthwhile. Therefore, monitoring methane as an indicator for ROS‐driven processes might be a promising approach. This might include the application of stable isotope labelling experiments (with an ^2^H and/or ^13^C label), as this approach could specifically visualise ROS‐related methane formation and thus overcome the problem of higher breath methane background levels derived from other sources as it was very recently shown for tobacco plants ^11^

This might be only half of the story as the formation of methane implicates the question as to whether this simple molecule might play a much broader, physiological role in aerobic life. Indeed, previous studies have demonstrated that non‐asphyxiating amounts of methane ameliorate the function of various tissue barriers and reduce the harmful biochemical and structural consequences of oxidative and nitrosative stress responses in pro‐inflammatory disease models.^12^, ^13^ However, so far there exists no experimental evidence for a biochemical degradation pathway of methane in aerobic organisms. Nevertheless, multiple lines of evidence also established that exogenous methane modulates key events of inflammation via master switches, such as Nrf2/Keap1 and NF‐κB, and influences mitochondrial respiratory capacity and critical components of ER‐mitochondria‐related pro‐apoptotic signalling pathways.^14^, ^15^ Another important aspect of methane bioactivity might be a potential interaction with other biological gases or gasotransmitters like NO, CO and H_2_S.^16^, ^17^ Thus, it is conceivable that cells have evolved means to utilise methane as a signalling molecule to trigger adaptive stress responses, which, in turn, could provide a rationale for the use of methane as therapeutic gas.^18^, ^19^ In this framework, the efficacy of experimental methane‐based therapies is rather well characterised in clinically relevant pulmonary stress conditions.^17^ Veno‐venous extracorporeal membrane oxygenation (vv‐ECMO) may provide adequate oxygenation for critically ill patients with pulmonary failure, but the life‐saving technique can lead to serious complications, partly due to the artificial gas exchange itself. The lung‐protective phenomena related to increased methane input imply that the effects of exogenous methane‐enriched gas mixtures should be examined in this direction, too. It is also of note that the cold storage in methane‐enriched organ preservation solution provided significantly increased defence against ER stress‐linked pro‐apoptotic signalling, mitochondrial damage and cardiac dysfunction during experimental heart transplantation as well.^20^ In future research, more disease models should be examined to confirm the dose‐dependent therapeutic effects of methane but also to investigate its degradation products.

For many years, methane has been considered physiologically inert in eukaryotic cells. However, the strong lines of evidence for formation and consumption processes strongly indicate a bioactive role of methane on a cellular level. An in‐depth understanding of the various factors that control cellular methane formation and consumption and a thorough understanding of its dual function and bioactivity may bring significant benefits to the domain of human health and disease. This might strongly stimulate biomedical research of endogenous and exogenous methane in the future.
